# Evidence of Mpox clade IIb infection in primary human alveolar epithelium

**DOI:** 10.1080/22221751.2025.2477845

**Published:** 2025-03-10

**Authors:** Thanaphon Namporn, Suwimon Manopwisedjaroen, Montien Ngodngamthaweesuk, Ekawat Pasomsub, Natnicha Jiravejchakul, Rattatammanoon Saengfak, Marea Jikka Nealiga, Arunsajee sea-be, Aalok Basu, Parichart Naruphontjirakul, Suradej Hongeng, Teresa D. Tetley, Arunee Thitithanyanont, Pakatip Ruenraroengsak

**Affiliations:** a Division of Pharmaceutical Technology, Department of Pharmacy, Faculty of Pharmacy, Mahidol University, Bangkok, Thailand; b Centre of Molecular Targeting and Integrated Drug Development (CMT-IDD), Faculty of Pharmacy, Mahidol University, Bangkok, Thailand; c Department of Microbiology, Faculty of Science, Mahidol University, Bangkok, Thailand; d CardioThoracic Surgery Unit, Department of Surgery, Faculty of Medicine Ramathibodi Hospital, Mahidol University, Bangkok, Thailand; e Virology Unit, Department of Pathology, Faculty of Medicine Ramathibodi Hospital, Mahidol University, Bangkok, Thailand; f Biological Engineering Program, Faculty of Engineering, King Mongkut’s University of Technology Thonburi, Bangkok, Thailand; g Department of Pediatrics, Faculty of Medicine Ramathibodi Hospital, Mahidol University, Bangkok, Thailand; h Lung Cell Biology, Section of Airways Disease, National Heart & Lung Institute, Imperial College London, London, UK

**Keywords:** Mpox, alveolar epithelial cells, lung model, air-liquid interface, pathogenesis, monkeypox virus, Orthopoxvirus

## Abstract

Monkeypox virus (Mpox) has been recognized for causing distinct skin lesions and is primarily transmitted through skin and sexual contact. To date, the transmissibility and pathogenesis of the Mpox virus in distal human lung has never been completely explored. Here the transmission pathways and Mpox tropism on patient-derived air-liquid epithelium (ALE) model fabricated using isolated primary human alveolar epithelial cells (hAECs) were investigated. hAECs were cultured and exposed to the Mpox virus clade IIb isolated from the patient. DNA, proteins, and the tropism were elucidated using polymerase chain reaction (PCR), Western blot, and high-content fluorescent imaging. Transmission electron microscopy (TEM) was employed to systematically observe the cellular distribution of viral particles. Viral titres were determined by TCID_50_ assay. Innate immune response and inflammatory mediators were measured using Milliplex® multiplex and ELISA analysis. Pathology at alveolar barrier integrity was determined using transepithelial electrical resistance (TEER) analysis. The study included mock-infected cells as control. Mpox virus significantly infected 42.82% of total hAEC populations. The prominent observed pathology included a significant reduction in TEER values, loss of tight junction protein, presence of tunnelling nanotubes (TNTs), and syncytium morphology. Four stages of Mpox biogenesis were clearly observed without significant activation of IL-6, MIP1alpha, TNF-α, and Galectin-9, although IL-1β were subtly promoted. The developed patient-derived ALE is a versatile model for Mpox virus clade IIb infection reflecting respiratory transmission competence of the Mpox. Postinfection lung pathogenesis demonstrated alveolar barrier damage without significant inflammation, raising concerns about possible immune evasion by the virus.

## Introduction

Since its identification in monkeys in 1958 and the first human case in 1970, monkeypox (Mpox) has been known for its dermatological symptoms particularly lymphadenopathy and rash lesions which concentrated on the face, distal extremities, and sexual organs [1]. Mpox belongs to the Poxviridae family and the Orthopoxvirus genus and currently has been classified into two clades. Clade I and Clade II, each subdivided into subclades Ia, Ib, IIa, and IIb [2]. While Clade Ia and IIa remain endemic in Africa with exclusively zoonotic transmission, clade IIb emerged from the West African lineage and has become the 2022 pandemic and linked to the recent global pandemic of Mpox virus [3,4]. Furthermore, a recently identified subclade Ib has been located outside of Africa, with instances recorded in Thailand, Sweden, Germany, and the UK [5–7], raising further concerns within the global health community, as clade I was proven to be more fatal and virulent than clade II [8]. As of Oct 15, 2024, 110,570 cases have been identified and 237 death cases have been reported worldwide [9].

Several *in vitro* and *in vivo* models have been used for studying Mpox susceptibility [10–12]. Although skin is the primary target [11], infectivity reports in animal models demonstrated transmission *via* intranasal and inhalation suggesting possible airborne transmission [12]. Therefore, concern on Mpox infectivity in human lung and its potential respiratory pathology has been raised. Recent clinical evidence came from patients who had positive PCR results of the nasal swabs and autopsy which accompanied by respiratory symptoms including cough, sore throat, dyspnea nasal congestion [13] and the evidence from the autopsy, computed tomography (CT), positron emission tomography/computed tomography (PET/CT), and chest X-ray which were pulmonary nodules, nodular consolidations, pleural effusion and reticular opacities, particularly in immunocompromised patients [14–18]. However, there is no evidence confirming the infectivity of the Mpox virus clade IIb in the human distal lung particularly at the airliquid alveolar epithelium layer – the first-line target of airborne transmission pathogens. Consequently, the current evidence of Clade IIb Mpox infection in human alveoli solely has relied on the A549 cell line which may not simulate the authentic phenotype of alveolar epithelial cells because of its deficiencies of the important identities such as lung surfactants and surfactant protein secretion [19,20]. Although stem cell-derived hAECs are regularly available, the derived hAECs cannot accurately replicate the function of isolated primary hAECs [21] that play a decisive role against pathogen infection [22]. Human alveoli are occupied by alveolar epithelial type 1 cells (hAEC1), alveolar epithelial type 2 cells (hAEC2), and alveolar macrophages (AMs). The hAEC1 inhabits ∼95% of the alveolar surface area and helps with gas exchange [23], while the hAEC2 inhabits the remaining ∼ 5% of the alveolar surface area and secretes lung surfactant to protect against lung collapse [24,25]. AMs involve in body homeostasis by removing foreign particles and microorganisms from the lungs. Neither of the previous studies have focused on Mpox tropism nor pathology within the human alveoli [8,26,27]. Therefore, it is crucially important to develop a robust patient-derived air-liquid epithelium model fabricated from hAEC to decipher this mystery. This result will help us to understand the persistence of the Mpox virus within the respiratory zone in human lungs as well as its potential to cause pulmonary complications.

This work is aimed to confirm if the Mpox has the ability to infect human distal lungs or not using patient derived ALE model and to explore its inflammatory consequences after the infection. Isolated hAECs have been employed to develop ALE model to resemble the actual alveolar architecture. Our pioneered work demonstrated that the developed ALE model robustly provides solid evidence of Mpox clade IIb virus infection and its pathogenesis in human alveoli, as well as potential use of the developed model for antiviral drug screening and exploring the interactome between pathogens and human alveolar cells.

## Methods

### Isolation, characterization, and fabrication of patient-derived air-liquid alveolar epithelium using primary hAECs

Procedure for tissue collection was approved by the Human Research Ethics Committee, Faculty of Medicine Ramathibodi Hospital (approval number: MURA2021/620). All methods were carried out in accordance with relevant guidelines and regulations. Primary hAECs were isolated based on our previous protocol [28]. Cells were divided into 2 sets. The first set of ∼50,000 cells were diluted in DCCM-1 media (Sartorius, Germany) containing 10% (v/v) serum, 4 µM GlutaMAX™, and 1% Antibiotic-Antimycotic (Gibco, US) and were seeded onto an apical side of the collagencoated transwell^TM^ for 1–3 days at 37°C and 5% CO_2_ until hAECs attached and covered all surface area of the cell culture membrane (90-95% confluence). Complete DCCM-1 media was filled in both sides of Transwell™ during the culture period. After cell attachment, the media were discarded from the apical chamber to establish an airliquid interface (ALI) culture before viral infection in BSL-3 on day 7. The media in the basolateral side was replaced every 3 days.

The second set of cells isolated from human lungs was cultured in a similar way as described for the first set, but the alkaline phosphatase (ALP) assay kit (Abcam, US) was used to characterize hAEC type 2 (hAEC2) and their transdifferentiation kinetics for the quality control process. Generally, the isolated cells are presented as hAEC2. As human progenitor cells, once they are attached to the membrane in an apical chamber of the transwell^TM^; they can transform into hAEC1. This in-house control set of cells was employed to confirm the ratio between hAEC1 and hAEC2 population (50%:50%) during the infection period. The ALP protocol of the assay kit is based on the manufacturer’s instructions. The presence of cell transdifferentiation was routinely measured before the Mpox virus inoculation. Briefly, isolated hAEC2 from individual donors was aimed to culture for 16 days in DCCM-1 media in the same condition as mentioned above. The percentage of alkaline phosphatase positive (ALP^+^) cells was assessed every 2 days until day 16 of the culture period. After fixing with 2.5% (w/v) paraformaldehyde in DPBS solution, equal volumes of the staining solution A and the staining solution B were mixed together and stained on the hAEC layer for 30 minutes incubation at 37°C and 5% CO_2_. The supernatant was discarded, and the cells were washed again with 1X DPBS two times and the sample was mounted using glycerol onto the microscope slides. The images were taken at 10x magnification using Olympus CKX53SF inverted microscope (Olympus, Japan).

### Mpox virus isolation, collection, infection, and viral titre analysis

The Mpox virus specimen was obtained from a leftover nasopharyngeal swab of a confirmed patient at Ramathibodi Hospital, Thailand, in late 2023, with ethical approval granted by the Human Research Ethics Committee (approval number: hMpxV/THA/V241-0053/2023). All methods were carried out in accordance with relevant guidelines and regulations. A leftover swab underwent isolation procedures beginning with filtration through a 0.45 µm filter. The filtrate sample was then cultured in Vero cells (ATCC, USA) in MEM containing 2% FBS, 100 units/mL, penicillin and 100 µg/mL streptomycin as previously described. By day 3, cytopathic effects were observed, leading to cell harvest and three freeze-thaw cycles [29]. Post-centrifugation, the supernatant was again filtered (0.45 µm) and propagated for three passages in Vero cells. Titration of the virus stock utilized plaque assays in Vero cells under a 1% agarose overlay in MEM with 2% FBS and 100 units/mL penicillin and 100 µg/mL streptomycin, visualizing plaques using crystal violet staining at day 6 post-infection. All procedures involving the live Mpox virus were performed in a certified biosafety level 3 facility, Faculty of Science, Mahidol University.

At 48 h post-infection (hpi), cell culture was observed for cytopathic effect under Nikon TS100 inverted microscope (Nikon, Japan) before cells and cell supernatants were collected for the viral titre analysis. Initially, hAECs were washed once with DPBS and then trypsinized for 10 minutes. The cell suspension was washed with PBS, and the cell pellet was resuspended in 100 µL/well before storing at – 80°C. Vero cells were used to determine viral titres, and they were plated in a 96-well plate and maintained at 37°C in the incubator with 5% CO_2_ overnight. The frozen hAEC samples were, then, thawed and subjected to three freeze-thaw cycles, as mentioned previously to release the virus from the infected cells [29]. The samples were then four-fold serially diluted in duplicated wells of a 96-well plate and allowed to be absorbed onto the Vero cell monolayer for 1 hour at 37°C in a 5% CO_2_ incubator. After absorption, the cell monolayer was washed once with 1X DPBS and then supplemented with MEM, 2% FBS, and antibiotics. The plate was further incubated at 37°C with 5% CO_2_ for 6 days. The virus titre, expressed as the 50% Tissue Culture Infectious Dose (TCID_50_), was determined by observing CPE on day 6. TCID_50_/mL was calculated with a TCID_50_ calculator based on the Reed-Munch method [30].

### Statistical analysis

Experiments with the isolated hAECs on ALE models were done independently using tissues from 11 different donors. The difference between infected and uninfected samples was analysed by the paired t-test for samples that passed the normality test, and the Wilcoxon matched-pairs signed rank test for samples that failed the normality test. Viral titres from different compartments of Transwell™ were analysed by a repeated measures ANOVA test applying Greenhouse-Geisser correction to account for potential violations of sphericity, followed by Tukey's multiple comparisons test. On the other hand, viral titres from the same compartment but compared between an infected sample and a control sample were analysed by Wilcoxon matched-pairs signed-rank test. hAEC transdifferentiation kinetics were analysed by Friedman test followed by the two-stage linear step-up procedure of Benjamini, Krieger, and Yekutieli for multiple comparisons, controlling the desired false discovery rate set at 0.05. Statistical analysis was performed using GraphPad Prism version 9.0.0. Statistical difference was considered at *P* < 0.05.

### Details of other methods

Due to word limitation, detail of other methods used in this experiment include Mpox viral sequencing and phylogenetic analysis, immunofluorescence imaging, alkaline phosphatase (ALP) assay for determining transdifferentiation kinetic of hAEC2, western blot, real-time PCR, assessment of innate immune response and inflammatory cytokines using Milliplex and ELISA, transmission electron microscopy (TEM) analysis, and transepithelial electrical resistance (TEER) were described in the supplementary material.

## Results

### Fabrication of patient derived air-liquid alveolar epithelium using primary hAEC

**Figure 1.**
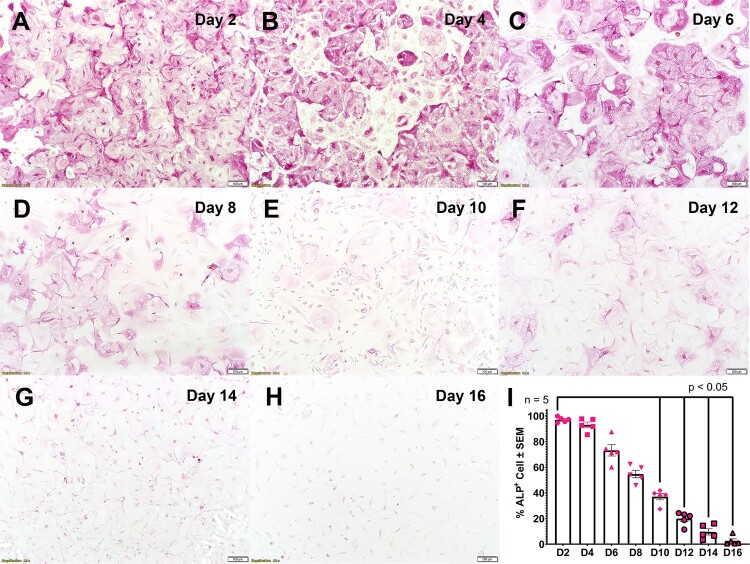
Transdifferentiation kinetic of hAEC2. Visualization of ALP positive cells by inverted light microscope at 10x magnification from Day 2 to Day 16 (A–H). Pinkstained represents ALP expression. Bar plot visualization of the percentages of ALPexpressed cells over 16 days (I). Data represents a mean percentage of ALP positive cells ± SEM, *n* = 5.

A model of patient-derived air-liquid alveolar epithelium model using primary hAEC cells has been successfully developed. This model allowed customization of the populations of hAEC1 and hAEC2 cells. Although, in the normal human lung, the ratio between the number of hAEC1 and hAEC2 is 1:1–1:2 [31,32]; we therefore selected a ratio at 1:1 (hAEC1:hAEC2) to provide an equal probability of Mpox infection for both cell types. The study of the in-house culture (quality control set) established the transdifferentiation kinetic of the hAEC2 into hAEC1 in the developed ALE model as showed by the significant decrease in ALP positive cells (*n* = 5, Figure 1(A–I)) at the longer culture period. The behaviour paralleled the significant decline in pro-surfactant protein C (Pro-SFTPC) expression (Figure S1(A–C)), whereas a significant increase in expression of caveolin-1 was observed suggesting the presence of hAEC1 in the model (Figure S1(D–F)). The major advantages of the developed model are the customization of cell population between hAEC1 and hAEC2 from the same donor, and the presence of a lung surfactant layer at the air-liquid interface within the model which genuinely replicates *in vivo* condition.

### Confirmed evidence of human alveoli susceptibility to Mpox clade IIb and pathogenesis

The consensus sequence of the Mpox culture isolates from nasal left-over specimen of a confirmed patient showed 100% genome coverage with an average coverage depth of 17,113.3x. Phylogenetic result of the Mpox genome revealed that the isolated virus used in the current study was within Lineage C.1, in clade IIb, which corresponds to the globally circulating clade IIb at the time of the sample collection (indicated with red lettering in Figure S1).

**Figure 2.**
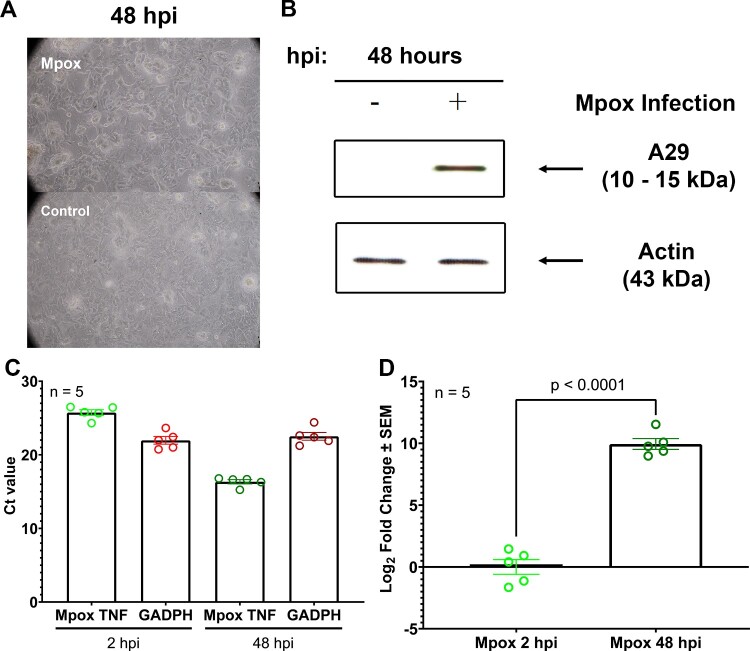
Confirm evidence of human alveoli susceptibility to Mpox in patient-derived ALE model at 2 and 48 hpi. (A) Cytopathic effect of Mpox on hAECs morphology. (B) Western blot of patient-derived ALE model infected with Mpox virus Clade IIb at 5 MOI, 48 hpi. (C) Ct value of Mpox TNF receptor normalized with GADPH as a housekeeping gene at 2 and 48 hpi. (D) Log_2_ fold change of Mpox TNF receptor at 2 and 48 hpi.

The confirmed evidence of human alveoli susceptibility to Mpox was analysed on a developed ALE model following 48 hours of viral inoculation. Baseline information of all donors was addressed in Table S1 comprising 7 females and 4 males and a mean age of 68 years old. The first 5 donor samples (no.1-5, Table S1) were employed for determining hAEC2 transdifferentiation kinetics. The rest (no.6-11, Table S1) were used for Mpox infection analysis. The results revealed a clear exhibition of cytopathic effect (CPE) across a layer of hAECs, characterized by cell rounding morphology, cell detachment, and cell death using light microscopy (Figure 2(A)). The evidence of Mpox infection and replication was also confirmed by western blot of protein A29 (Figure 2(B)) and rt-PCR of Mpox TNF gene at 2 and 48 hours (Figure 2(C, D)).

**Figure 3.**
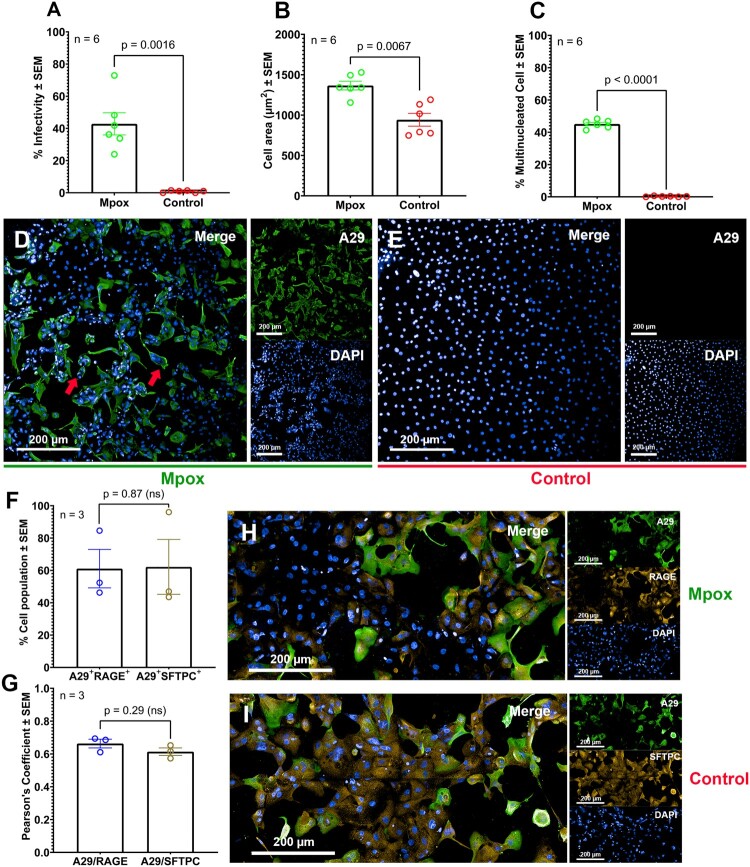
Mpox pathogenesis and Mpox tropism in patient-derived ALE model at 5 MOI, 48 hpi. (A) Mpox infectivity, (B) cell size comparison between infected and non-infected cells, (C) percentages of multinucleated cells observed in infected and non-infected cells, (D-E) immunofluorescent imaging of A29-positive cells in infected and control samples, (F) colocalization analysis of A29 against RAGE or SFTPC, (G) high content imaging analysis of A29+RAGE+ cells or A29+pro-SFTPC+ cells. Data represents a mean percentage of cell population ± SEM from 16 individual images of cells on the Transwell™ at 10x magnification. (H-I) Immunostaining images of A29 against RAGE or pro-SFTPC. A29 (Mpox; Green), RAGE or pro-SFTPC (hAEC1 and hAEC2 marker, respectively; Orange), and DAPI (Nuclei, Blue). The image was taken at 63x Magnification, scale bar at the bottom.

For the analysis of Mpox pathogenesis, 42.82 ± 6.88% of hAECs were infected compared with the control (Figure 3(A)). Additionally, the formation of syncytia characteristics in hAECs had simultaneously been observed in Mpox-infected samples where the infected cells were agglomerating and enlarging. High content analysis results illustrated a significant increase in cell surface area from 942.63 ± 80.03 µm^2^ (noninfected cells) to 1366.17 ± 54.55 µm^2^ (infected cells, Figure 3(B)). Within 42.82% of the infected cells, 45.13 ± 1.06% of these cells were found to exhibit multinucleated character or “syncytia structure” (Figure 3(C, D)) while this structure was not observed in the control (Figure 3(E)). We also found that the cell nuclei of the infected cells appeared to lose their intact shape and formed an unusual structure. Overall examination of the entire epithelium layer revealed a disruption of the monolayer identified by the leakage of cell culture media from the basolateral side towards the apical surface.

To determine the tropism of the Mpox virus in alveolar epithelial cells, the colocalization analysis was utilized to investigate the spatial association between the viral A29 protein and cellular markers of hAEC1 (RAGE) /hAEC2 (SFTPC). Pearson's coefficients indicated substantial colocalization for both A29-RAGE (0.6636) and A29SFTPC (0.6140). However, no significant difference between these Pearson's coefficients was observed (*p* = 0.2854) (Figure 3(F)). Analysis from high-content screening also revealed a high degree of colocalization between the A29 and both markers, suggesting that both cell types have comparable infection levels (61.10% vs 62.19%, Figure 3(G)). This result corresponds to the observation from the immunofluorescent analysis in Figure 2(H, I), indicating that the Mpox virus exhibited no preference for infection between hAEC1 and hAEC2.

### Ultrastructure analysis inside hAEC

**Figure 4.**
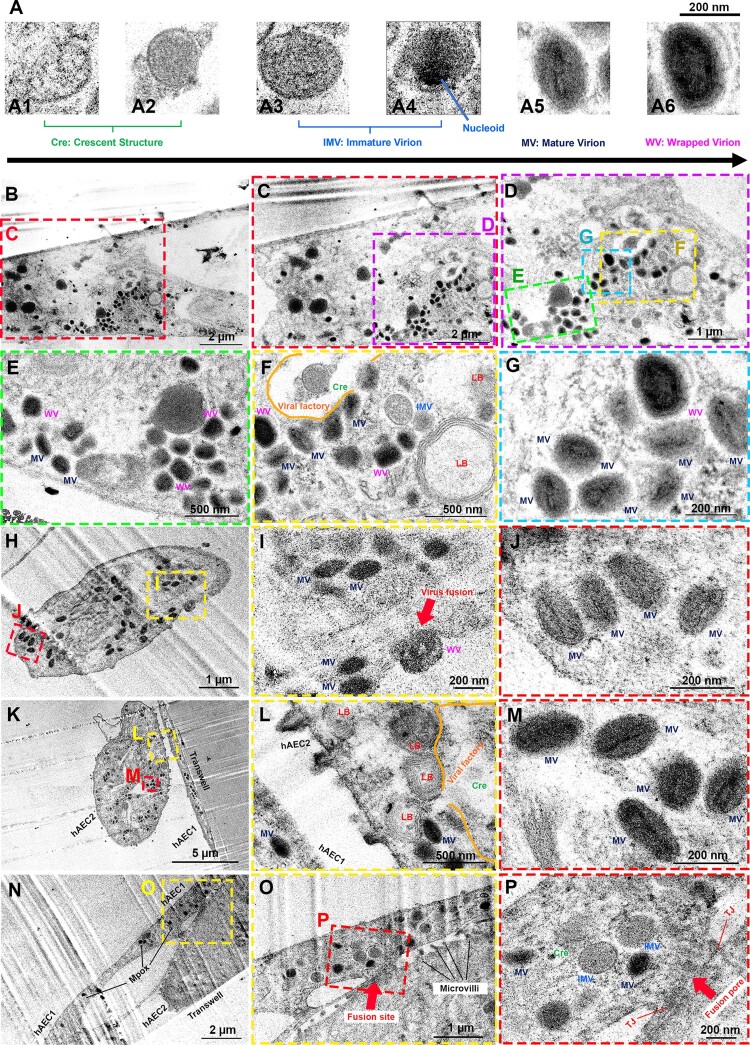
TEM image analysis of Mpox biogenesis in primary human air-liquid alveolar epithelium constructed from hAECs. (A) TEM images visualized different forms of Mpox virus showing Mpox biogenesis in hAEC at 5 MOI, 48 hpi: (A1, A2) Mpox’s crescent structure, (A3) immature virion, (A4) the immature virion with visible nucleoid, (A5) the mature virion with a brick-shaped core and (A6) the wrapped virion with a double layer of a lipid bilayer. The scale bar at the top in Figure 4(A) is 200 nm. (B) Different regions of Mpox tropism at low magnification. (C) High magnification of the red square in Figure 4(B) represents hAEC2 infected cell and (D) the higher magnification of the purple square in Figure 4(C). (E-G) Mpox tropism within human alveolar epithelial. The green, turquoise, and yellow squares in Figure 4(D) highlight the presence of the mature and wrapped virions (E–G) as well as the crescent structure and the immature virion (F) in hAEC2 cell. (H) Mpox virus fusion with the host cell plasma membrane was clearly observed and (I) the high magnification of the yellow square in Figure 4(H) shows the viral and host cell membrane fusion process. (J) High magnification of the red square in Figure 4(H) shows intracellular Mpox virions at mature status. (K) Mpox tropism within hAEC1 and hAEC2 cells. (L) High magnification of the yellow square in Figure 4(K) shows the ultrastructure of lamellar bodies and the area of the viral factory in hAEC2. (M) High magnification of the red square in Figure 4(K) shows mature Mpox virions. (N) Syncytium formation between infected hAEC1 and infected hAEC2. (O) Selected regions at higher magnification from Figure 3(N) revealed a fusion site between hAEC1 cell and microvilli of hAEC2 cell where the tight junction (P) was observed. Scale bars between 200 nm–2 µm. Cre = Crescent Structure. IV = immature virion. MV = mature virion. WV = wrapped virion. LB = lamellar body. TJ = tight junction. We also depicted the illustration of Mpox life cycle through our developed patient-derived air-liquid epithelium (ALE) model as can be seen in Figure S3.

Further insights were gained through TEM analysis, which delineated the biogenesis of the Mpox lifecycle within 24 h infected period including (i) Crescent Structure – the earliest identifiable viral structure that self-assembles into a crescent-shaped membrane (Figure 4(A1, A2)); (ii) Immature Virion – the previous crescent structure gradually transformed into a full spherical shape (Figure 4(A3)) before the viral DNA becomes more concentrated within the enclosed area and forming a visible nucleoid (Figure 4(A4)); (iii) Mature Virion – the stage where the viral genome is tightly condensed into brick or dumbbell structures in the core and flanked by two lateral bodies (Figure 4(A5)); and (iv) Wrapped Virion – one of the mature virion forms that has been wrapped at the Golgi compartment (Figure 4(A6)). The intracellular Mpox particles found were essential for cell-to-cell transmission. These stages involved early replication sites, maturation sites, and enveloped virions providing an in-depth understanding of the virus-host intracellular behaviour and lifecycle progression within both hAEC1 and hAEC2. Since the developed ALE model helped to preserve authentic features of hAECs and their interconnection and communication, we found all stages of Mpox particles distributed throughout the cytosol (Figure 4(B–G)). Figure 4(H–J) illustrated that the wrapped virion of Mpox virus could enter the cell by fusion through cellular membranes, facilitating the delivery of viral genetic material into the hAECs. Similar trends of Mpox infection were observed in both types of hAECs using TEM. This finding supports the previous evidence from immunofluorescence imaging that Mpox can infect both cell types with no selectivity (Figure 4(K–M)). This implied a broad cellular tropism for Mpox in distal human lungs. Furthermore, cell syncytium between hAEC type 1 and type 2 cells was shown as the formation of a fusion site between two cells as depicted in Figure 4(N–P). Tight junction was reduced in the infected sample and the forming of a fusion pore that allowed the transportation of Mpox virus between each cell was observed.

### Viral titre and cytokine response

**Figure 5.**
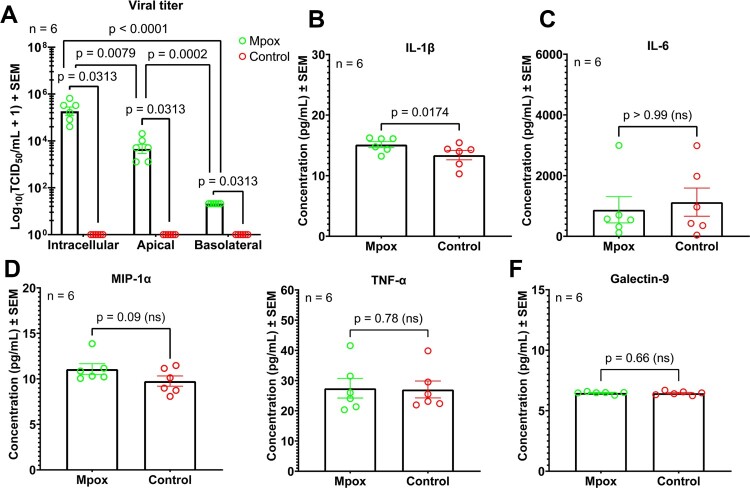
Mpox titre and cytokines, chemokines, and galectin-9 from apical chamber of the Transwell™ in Mpox-infected and control ALE model at 5 MOI, 48 hpi. (A) Mpox titre, (B) IL-1β, (C) IL-6, (D) MIP-1α, (E) TNF-α and (F) Galectin-9. Data represents a mean Log_10(_TCID_50_/mL + 1) ± SEM for viral titre and represents a mean concentration (pg/mL) ± SEM for the level of cytokines and chemokines, six replicates (*n* = 6).

Viral titre analysis from 3 sampling positions of the Transwell^TM^ demonstrated the highest number of viral particles inside the cell followed by the viral particles in the apical and basolateral sides, respectively (Figure 5(A)). This result suggested that Mpox cloud robustly replicates inside the cell more than being released into the extracellular space and preferentially budding into the apical side rather than the basolateral side. Although the Mpox titre was established, there were no significant differences (*p* > 0.05) in the levels of inflammatory mediators including IL-6, MIP-1α, TNF-α, and galectin-9 between infected and control cultures (Figure 5(B–F)). However, significant was observed with the level of IL-1β. Therefore, these findings suggested that Mpox clade IIb infection in patient-derived ALE model may not trigger a broad pro-inflammatory cytokine response as measured by IL-6, TNF-α, and MIP-1α, although IL-1β were subtly elevated (mean difference = 3.310 pg/mL, *p* = 0.0174). Galectin-9 levels, which can be potentially involved in an immune response against viral infection, also did not show a significant change upon Mpox virus infection.

### Compromising of alveolar barrier integrity during Mpox infection

**Figure 6.**
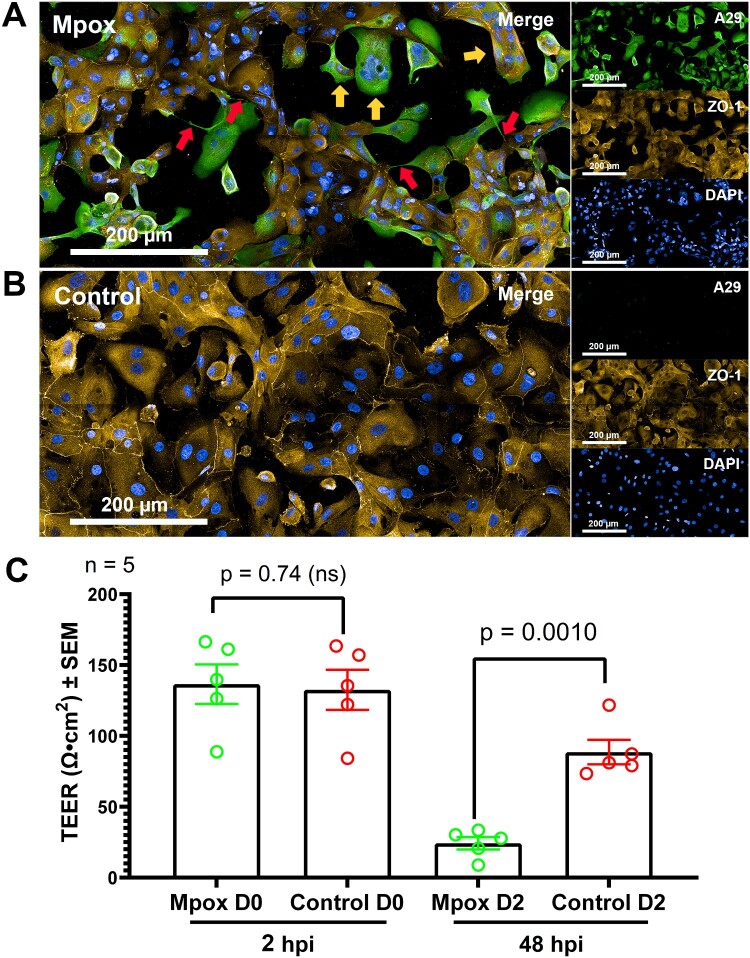
Alveolar epithelial barrier analysis in hAEC ALI culture at 5 MOI, 48 hpi. (A) Immunostaining images of Mpox-infected sample (B) and control sample. A29 (Mpox; Green), ZO-1 (Tight junction; Yellow and DAPI (Nuclei, Blue). The image was taken at 63x Magnification, scale bar at the bottom. Red arrows indicate the TNTs between cells. Yellow arrows indicate cell syncytium character. TEER analysis comparing Mpox-infected sample and control sample (C). Data are presented as means of TEER value (Ω·cm^2^) ± SEM with five replicates (*n* = 5).

Mpox infection decreased the expression of ZO-1 at the tight junctions (yellow in Figure 6(A, B)) along with the disruption of the alveolar epithelium layer as evident in Figure 6(A) compared with the control non-infected sample in Figure 6(B). This led to a weakening of the barrier of human alveoli. In addition, the TEER analysis provided insight into the integrity and permeability of cell monolayers in the air-liquid human alveolar epithelium model. Initially, both Mpox infected, and control non-treated samples exhibited similar TEER values of 132–137 Ω·cm², indicating healthy and intact epithelium barriers. Over the course of the infection for 48 hours, a significant decline of TEER values was observed in the Mpox-infected samples, the TEER was dropped to 24.22 ± 4.37 Ω·cm². In contrast, the control samples maintained a TEER value of around 88.56 ± 8.59 Ω·cm² (*n* = 5), indicating that the integrity of the epithelial barrier remained largely intact in the absence of viral infection (Figure 6(C)). The decrease in TEER by 82.25% in the infected samples from 136.49 Ω·cm² is a direct consequence of viral pathogenicity. In summary, here we provide systematic observation of immunostaining, western blot, TEM and TEER analysis indicates that Mpox virus infection may lead to damage at the air-liquid alveolar epithelium barrier.

Our experiment also observed the unique behaviour of the infected hAECs, where they appear to extend towards the neighbour cells and create tunnelling nanotubes (TNTs, red arrow in Figure 6(A)). We found that TNTs can facilitate cell-to-cell transmission of the intercellular Mpox virions, as visualized by the fluorescent A29 protein along these interconnections in Figure 6(A) (yellow arrows). Nonetheless, TNT is absent in the control non-treated group. This is the first time we observed TNT formation in the hAECs initiated by Mpox virus infection.

## Discussion

Most of the previous *in vitro* human distal lung models recruited human lung cancer cells to study host-pathogen interactions. However, the limitation of this approach lies in the genotype disparity between the human lung cancer cell lines and the isolated patient-derived hAECs [20]. The approach of using the induced pluripotent stem cell (iPSC) to derive alveolar epithelial cells (iAECs) needs to be standardized and provides mature alveolar epithelial cells at the desired number. In fact, when comparing barrier function and expression of alveolar epithelial cell markers between cultured iAEC and hAEC, the hAEC exhibited a greater degree of alveolar maturity than iAEC. This is making hAECs a reliable model for studying alveolar epithelium pathology [33]. In addition, since hAEC2s can transdifferentiate into hAEC1s *in vitro*, we revealed the transdifferentiating kinetics over a 16-day culture period to find a point where both cell types were present in relatively equal proportions. This was important for our Mpox infection experiments, as hAEC1 and hAEC2 cells may have different susceptibility to the virus and found that hAEC on around 8 days offered an equal proportion of both cell types. We, therefore, selected this time point for studying the Mpox infection in the patient-derived ALE model. Importantly, the environment of the developed ALE model could initiate *in vivo* physiological conditions than that of the previous 2D and 3D models [34]. Furthermore, the developed ALE model can offer a tight junction barrier, lung surfactant layer [19], and a stable alveolar maturity at the comparable level to *in vivo* conditions where the apical side is exposed to air and the basolateral side is exposed to the liquid medium. Our study clearly demonstrated that patient-derived ALE model can be used to study the transmission, and pathogenesis of Mpox virus by observing cytopathic characteristics (e.g. cell rounding, detachment, syncytium, and death). These findings were confirmed by both light microscopy, confocal microscopy, and immunohistochemistry. The presence of the Mpox virus was qualitatively and quantitatively analysed by qPCR, western blot, and TEM. Results also supported the potential transmission *via* the airborne through the presence of viable Mpox virus in the saliva droplets or the aerosols in the air by recent studies from the UK and Spain [35–37] and from the primate models [38–40]. Moreover, our results also illustrated the risk of alveolar layer disruption by Mpox infection.

**Figure 7.**
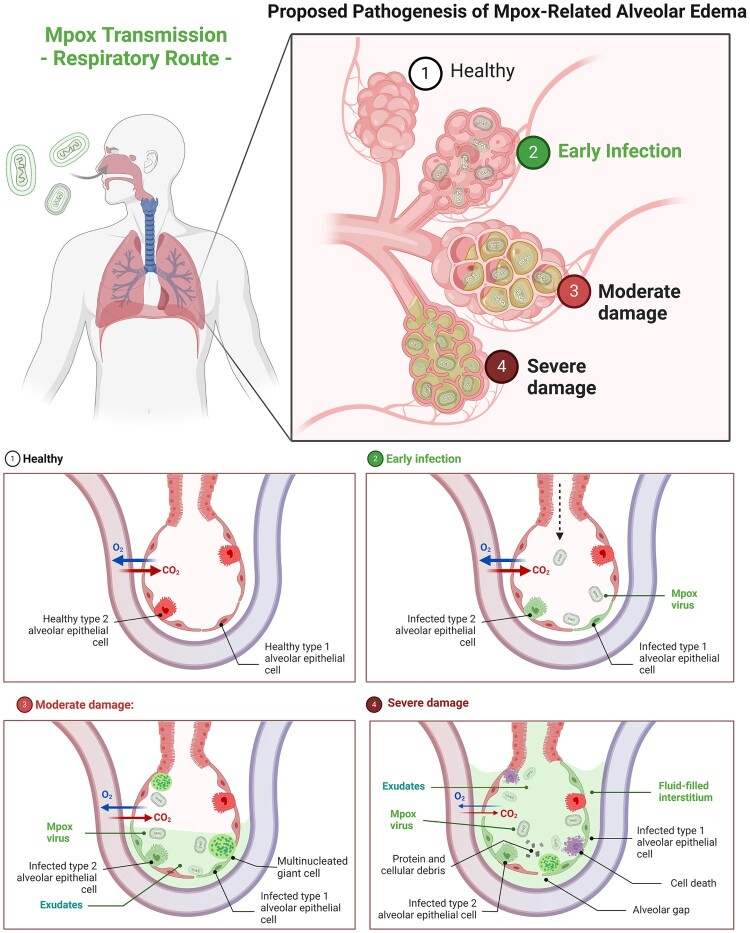
Proposed pathogenesis of Mpox-related alveolar oedema. Healthy State (Panel 1): The alveolar structure remains in a healthy state. Early Infection State (Panel 2): The Mpox begins to infect the alveolar epithelial cells without any noticeable disruption in gas exchange. Moderate Damage State (Panel 3): The infection progresses to moderate damage within the human alveoli. The presence of multinucleated cells, an indicative of viral-induced syncytium, and exudates start to accumulate in the alveolar space with a noticeable impairment in the gas exchange. Severe Damage State (Panel 4): Accumulated alveolar-resident cell death results in the formation of alveolar gaps and extensive pleural effusion, causing critical respiratory failure.

In human alveoli, hAEC1 and hAEC2 play different key roles. The hAEC1 acts as a gas exchanger and prevents the leakage of fluid from blood vessels and interstitial space into the alveolar sac. The hAEC2, on the other hand, acts as a lung surfactant factory which is extremely crucial for maintaining surface tension at the air-liquid interface, as well as, preventing fluid accumulation in the alveoli. Interestingly, hAECs displayed a low Mpox infection rate, suggesting that hAECs may possess some level of innate immunity protective role for Mpox infection. The presence of body homeostasis, including the secretion of lung surfactants and the production of surfactant proteins by hAEC2 may be one of the reasons for the low level of Mpox infection [22]. This finding emphasizes the importance of using relevant cell models, the patient-derived ALE model, to accurately assess Mpox infectivity and pathogenesis. We, next, explored Mpox tropism inside human alveoli by comparing hAEC1 and hAEC2 derived from the same donor, and we found no specific preference between both cells. Connecting the dots, our finding aligns with the clinical observations from autopsy [14], CT, and chest X-ray [15,18] of fatal Mpox patients and may potentially help explain the mechanisms. The death was reported to be initiated by extensive cell death and disruption of the alveolar epithelium layer together with the disruption of adjacent blood capillaries which led to exudative pleural effusion. Cell death and syncytium formation in human alveoli could increase permeability as evidenced by a significant reduction of TEER value and the loss of tight junction protein, allowing fluid accumulation within the alveolar sacs, and potentially contributing to alveolar oedema as a last resort (Figure 7).

Results revealed significantly higher viral titre within the infected cells compared to both the apical and basolateral supernatants. This data implied that the Mpox virus replicates robustly intracellularly, with less efficient release into the surrounding media. The apical supernatant exhibited the second-greatest viral titre, indicating that the virus was preferentially released towards the airway surface. This is consistent with the behaviour of many respiratory viruses, which infect and shed from the apical surface of airway epithelial cells, allowing airborne transmission by coughing or sneezing [41,42].

However, our findings showed no significant change in pro-inflammatory cytokine levels (IL-6, MIP-1α, TNF-α, and Galactin-9) in Mpox-infected cells compared to controls. Only the IL-1β was subtly increased. The lack of cytokine response points to the potential to evade early immune defenses in the distal lung of the Mpox virus. This immune evasion could challenge the diagnosis and hinder the event of Mpox-related alveoli damage. However, this evidence is contradictory to the previous studies that reported significant elevation of IL-6, MIP-1α, and TNF-α after the Mpox infection. This discrepancy might be attributed to the differences in the clade of the Mpox virus and the source of materials used for cytokine measurement where our study utilized cell supernatants from hAEC ALI cultures, and the previous research groups employed patient serum samples [43,44]. Moreover, we hypothesize that the indifferent levels of pro-inflammatory cytokines between the control and infected cells could be a direct consequence of multiple cell-to-cell transmission mechanisms that Mpox utilized for immune evasion.

Our results clearly demonstrated that the Mpox virions could cumulatively replicate within the infected cell and infect the nearby cells in human alveoli by releasing mature virion only from the disrupted infected cell or by the budding wrapped virion from the host cell. These can minimize their exposure time to the extracellular environment, as the mature virion will quickly infect adjacent cells, and the wrapped virion can be disguised in the host-derived additional membrane. Importantly, the genome and proteins of Mpox virion can be transported between each cell through the TNT. Intracellular Mpox contents that transport across this projection can evade the immune response by circumventing the immune system in the extracellular environment. This ultrastructure, therefore, offers two additional advantages to viruses. Firstly, this structure may allow the virus to easily infect the non – or less-permissive cells as demonstrated before with SARs-CoV-2, HIV, and influenza by circumventing the receptor-mediated entry.

Secondly, TNTs may also facilitate the spread of viruses in the presence of drugs and neutralizing antibodies [45,46]. The new finding mechanism is that the occurrence of syncytium formation may promote cell-to-cell or transcellular infection. Unlike TNTs, the typical syncytium process could fuse cells into a single entity accelerating viral infection. This process involves fusogenic proteins on the surface of the infected cells binding to receptors on adjacent uninfected cells. Upon receptor binding, the fusogenic proteins can undergo a conformational change and bring the membranes of the infected and target cells into proximity. Such a process destabilizes the membrane bilayers and facilitates cell fusion [47]. Although the result from cell syncytium formation by Mpox virus has not yet been fully understood, we hypothesized that Mpox virus may initialize this phenomenon for the energy distribution among the fused cells. This could create a more favourable environment for robust viral replication and potentially prepare the virus for extrapulmonary invasion. Further study on the presence of syncytium and TNTs in Mpox-infected cells may provide the reason why Mpox expresses no specific favourite to infect a specific type of hAEC. However, our study is limited by a number of the obtained isolated cells which did not allow us to perform the time-course analysis nor the cocultured immune response analysis. Thus, additional study on these subjects will help us to understand Mpox replication kinetics in hAECs as well as the protection against Mpox on hAEC.

In conclusion, we developed a robust model of primary human air-liquid alveolar epithelium model for Mpox pathological study and tropism. Our findings provide strong evidence supporting the potential respiratory transmission of Mpox virus clade IIb beyond the skin and traditional sexual transmission routes using patient-derived ALE model. Additionally, this study also sparks a discussion on how the evolution of the Mpox virus could alter its pathogenesis and enhance its transmission globally [48]. Recent evidence supported such evolution including the report of the sexual transmission of Mpox Clade Ib which was once believed to be initiated only by Clade IIb [49]. Further research is warranted. Exploration of the Mpox infection and shedding in different lung compartments and Mpox aerosol stability will be important in refining our understanding of the Mpox respiratory transmission dynamic.

## Supplementary Material

Figure S3.tif

Supplementary.pdf

Figure S1.tif

Figure S2.tif

## Data Availability

The complete genome sequence of Mpox Clade IIb strain hMpxV/THA/V241-0052/2023 has been deposited in GenBank under accession number PQ153226.
