# Supplementary figures and images for: Evidence of Mpox clade IIb infection in primary human alveolar epithelium
Source: Emerg Microbes Infect. 2025 Mar 10;14(1):2477845. doi: 10.1080/22221751.2025.2477845 (PMC11938317; doi:10.1080/22221751.2025.2477845)

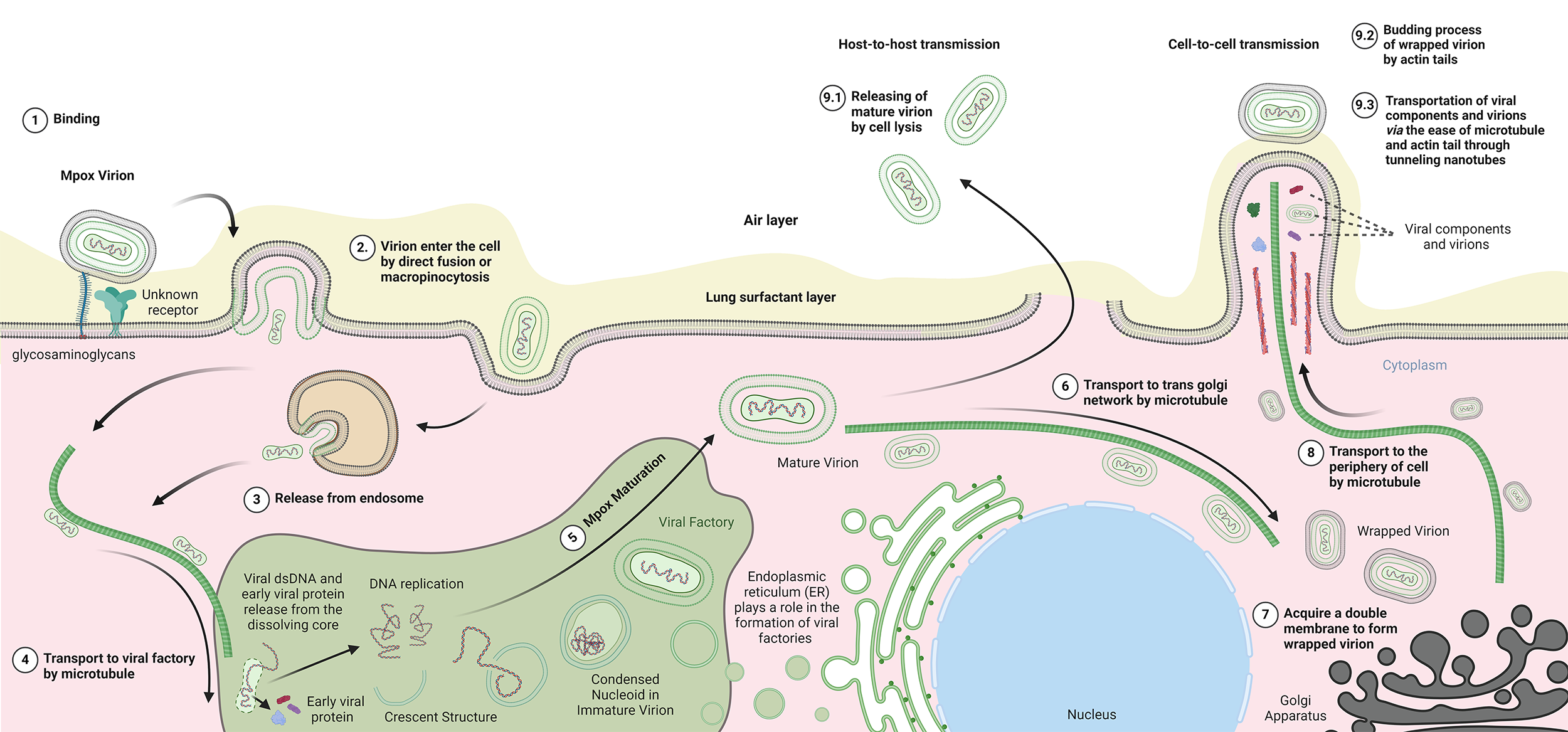

Supplement: Figure S3.tif [file TEMI_A_2477845_SM0820.tif]

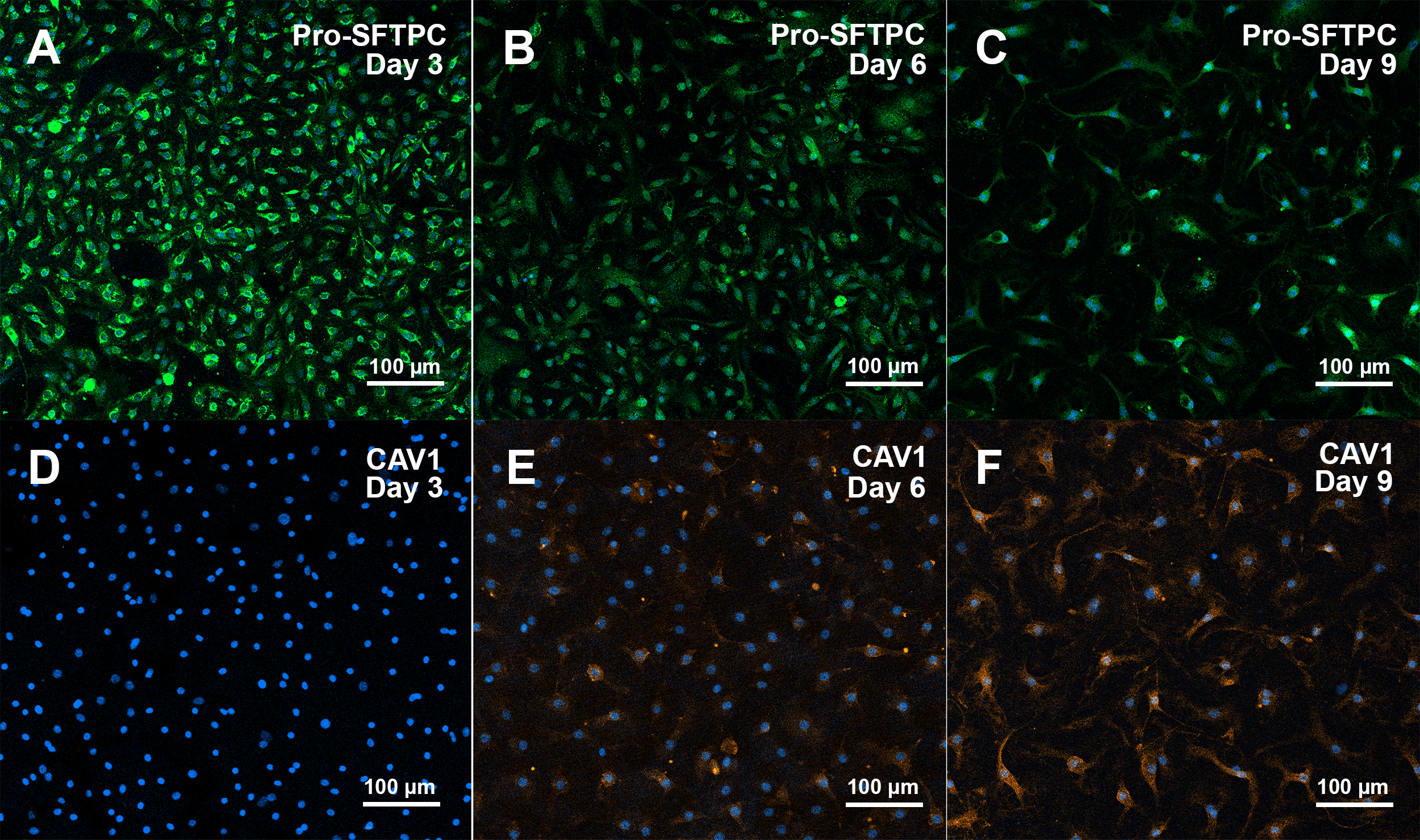

Supplement: Figure S1.tif [file TEMI_A_2477845_SM0818.tif]

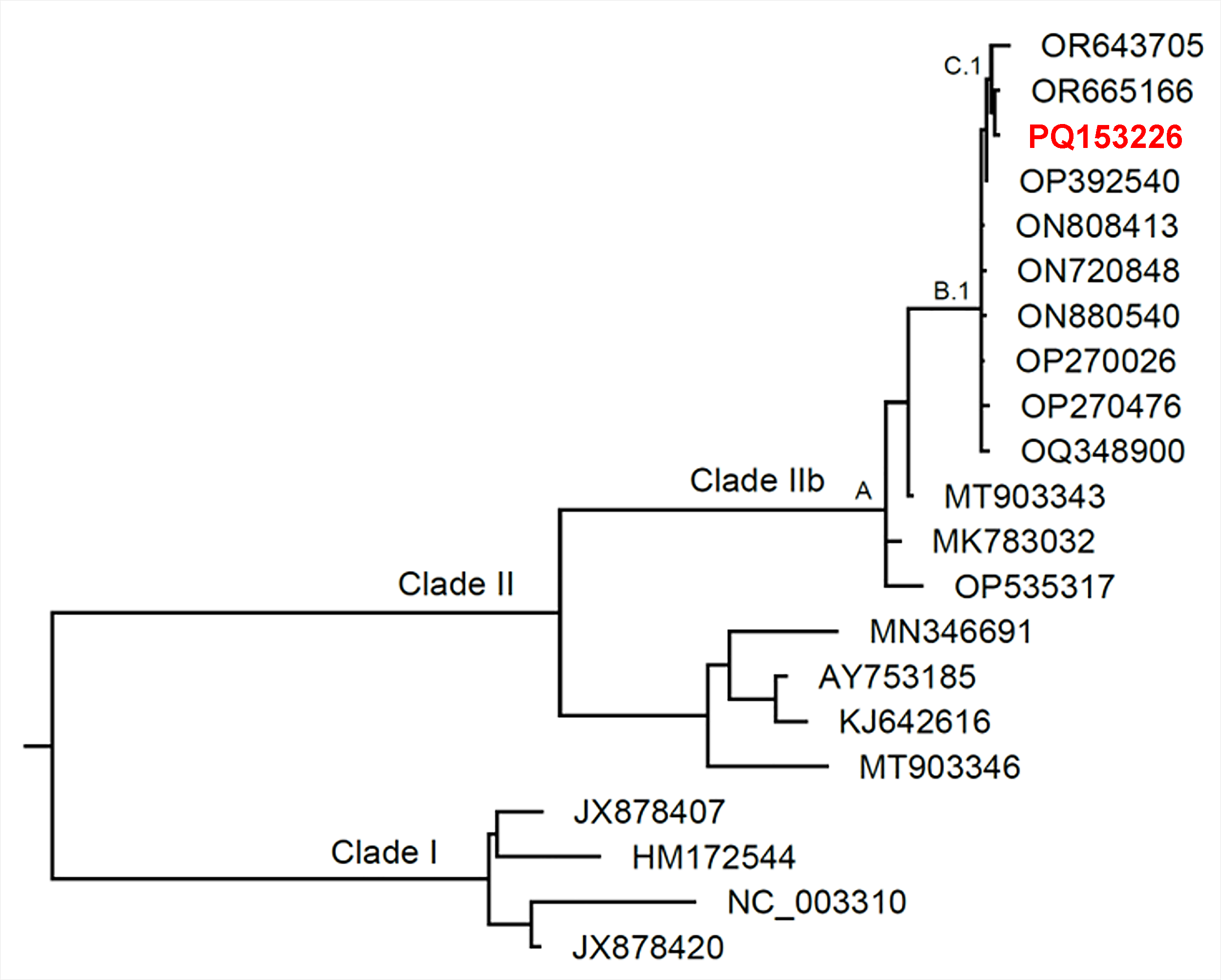

Supplement: Figure S2.tif [file TEMI_A_2477845_SM0817.tif]
