# Supplementary material for: Evidence of Mpox clade IIb infection in primary human alveolar epithelium
Source: Emerg Microbes Infect. 2025 Mar 10;14(1):2477845. doi: 10.1080/22221751.2025.2477845 (PMC11938317; doi:10.1080/22221751.2025.2477845)
Supplement: Supplementary.pdf [file TEMI_A_2477845_SM0819.pdf]

Supplemental Materials

Evidence of Mpox Clade IIb Infection in Primary Human Alveolar Epithelium

Thanaphon Namporn[a,b], Suwimon Manopwisedjaroen[c], Montien Ngodngamthaweesuk[d], Ekawat Pasomsub[e], Natnicha Jiravejchakul[c], Rattatammanoon Saengfak[c], Marea Jikka Nealiga[c], Arunsajee Sea-be[a,b], Aalok Basu[a,b], Parichart Naruphontjirakul[f], Suradej Hongeng[g,i], Teresa D Tetley[h,i], Arunee Thitithanyanont[c]*, Pakatip Ruenraroengsak[a,b]*

[a]*Division of Pharmaceutical Technology, Department of Pharmacy, Faculty of Pharmacy, Mahidol University, Bangkok, Thailand 10400*

[b]*Centre of Molecular Targeting and Integrated Drug Development (CMT-IDD), Faculty of Pharmacy, Mahidol University, Bangkok, Thailand 10400*

[c]*Department of Microbiology, Faculty of Science, Mahidol University, Bangkok, Thailand 10400*

[d]*CardioThoracic Surgery Unit, Department of Surgery, Faculty of Medicine Ramathibodi Hospital, Mahidol University, Bangkok, Thailand 10400*

[e]*Virology Unit, Department of Pathology, Faculty of Medicine Ramathibodi Hospital, Mahidol University, Bangkok, Thailand 10400*

[f]*Biological Engineering Program, Faculty of Engineering, King Mongkut's University of Technology Thonburi, Bangkok, Thailand 10140*

Department of Pediatrics, Faculty of Medicine Ramathibodi Hospital, Mahidol University, Bangkok, Thailand 10400

[h]*Lung Cell Biology, Section of Airways Disease, National Heart & Lung Institute, Imperial College London, Sir Alexander Fleming Building, South Kensington Campus, London, UK, SW7 2AZ*

[i]Essentially Intellectual Contributor

*Correspondence: Assoc. Prof. Dr. Arunee Thitithanyanont (Email: arunee.thi@mahidol.edu) and Assoc. Prof. Dr Pakatip Ruenraroengsak (Email: pakatip.rue@mahidol.ac.th)

Contributors: PR designed and coordinated the study on patient-derived ALE model development and viral exposure parts, analyzed and interpreted the results. AT designed and coordinated the study, analyzed and interpreted the results of viral exposure part. MN and SH provided the subject donor sample of the lung tissue. EP conducted nasal swab samples, SM isolated Mpox virus. TN isolated hAEC2 cells from subject donor samples, and TN, AS, PR developed patient derived ALE model. SM performed viral exposure and $TCID_{50}$ assay. TN performed viral exposure, TEER evaluation, Milliplex, ELISA and immunofluorescent imaging using high-content imaging. TN, AB and PN processed TEM samples and performed TEM analysis. RS conducted western blot analysis, and MJN conducted PCR analysis. NJ performed viral sequencing and phylogenetic analysis. TN, SM, RS, MJN and NJ performed, analyzed and interpreted experimental data. TT provided technically supported hAEC2 cells and ALE development. The manuscript was substantially contributed by PR and AT. PR, AT, SH and TT critically reviewed the manuscript. All authors have discussed the results, commented, and agreed to the published version of the manuscript.

Detail Methods

Mpox viral sequencing and phylogenetic analysis

Total nucleic acid was extracted from the culture-passaged Mpox isolate using the GENTi™ Advanced Viral DNA/RNA Extraction Kit (GeneAll Biotechnology, Korea), and further used for library preparation with the KAPA HyperPlus kit (Roche, Switzerland). The library was subsequently sequenced with the Illumina paired-end sequencing, 2 x 150 bp (Macrogen, Korea), yielding a total output of approximately 130M reads. Raw FASTQ files were filtered using Fastp [1] to retain reads with a mean base quality score higher than 30 and a minimum read length of 70 bp, using a sliding window of 4 bases. The cleaned data were aligned to Mpox reference genome NC_063383.1 using BWA [2] with an aligned read quality of 50. SAMtools [3] was then used to sort and index a BAM file. Finally, a consensus sequence of Mpox was generated with iVar [4], using a q score greater than 20 and a depth greater than 50. The consensus sequence was further used to perform phylogenetic analysis using the NextClade tool V3.3.1 [5] (https://clades.nextstrain.org/), and the phylogenetic tree was visualized with FigTree v1.4.4 (http://tree.bio.ed.ac.uk/software/figtree/). The complete genome sequence of Mpox virus has been deposited in GenBank under accession number PQ153226.

Immunofluorescence imaging

The infected and non-infected hAECs were fixed with 3.7% (v/v) paraformaldehyde solution for 20 minutes, then permeabilized with 0.1% Triton-X in DPBS for 5 minutes and washed several times with DPBS for 5 minutes each. Then, the samples were incubated with a blocking buffer containing 1% w/v bovine serum albumin and 0.01% (v/v) Tween 20 for 30 minutes at room temperature (25°C). The hAEC layer was sequentially stained with Mpox virus rabbit polyclonal A29 antibody (Sino Biological,

China) at a concentration of 1:200 overnight at 4°C. For identifying hAEC type 1 (hAEC1) or type 2 (hAEC2) mouse monoclonal surfactant protein c (SFTPC) antibody (Santa Cruz Biotechnology, US) and mouse monoclonal receptor for advanced glycosylation end products (RAGE) antibody (Santa Cruz Biotechnology, US) at a concentration of 1:250 were used and stained in the same condition as A29 antibody. After 24 h, cells were rinsed with DPBS for 5 minutes 3 times before being tagged with secondary immunofluorescence Alexa Fluor™ 488 and Alexa Fluor™ 555 antibodies for 1 hour at room temperature in the dark condition. The samples were rinsed and mounted with Fluoroshield Mounting Medium with DAPI (Abcam, US). All immunofluorescent confocal images were taken using confocal images by Opera Phenix Plus high-content screening system (PerkinElmer, US). Additional methodology of high-content image analysis for determining Mpox virus infectivity, cell area, number of multinucleated cells in the Mpox infected population, and others were analyzed using Harmony software version 5.2 as mentioned in Table S2.

High-content image analysis

Alveolar epithelial cell Mpox infection was assessed *via* high-content screening using 16 maximum-projection image sections from transwell membranes, encompassing 80% of the culture area. Images were analyzed in Harmony software version 5.2 with the following analytic sequences in Table S2. Then, the results were collected and analyzed using GraphPad version 9.0.0.

Alkaline phosphatase (ALP) assay for determining transdifferentiartion kinetic assay of hAEC2

Alkaline phosphatase (ALP) assay kit (Abcam, US) was used to characterize hAEC type 2 (hAEC2) and their transdifferentiation kinetics. The protocol is based on the

manufacturer's instructions. Briefly, isolated hAEC2 from individual donors was aimed at culture for 16 days in DCCM-1 media in the same condition as mentioned above. The percentage of alkaline phosphatase positive (ALP+) cells was assessed every 2 days until day 16 of the culture period. After fixing with 3.7% (w/v) paraformaldehyde in DPBS solution, equal volumes of the staining solution A and the staining solution B were mixed together and stained on the hAEC layer for 30 minutes incubation at 37°C and 5% CO2. The supernatant was discarded, and the cells were washed again with 1X DPBS two times and the sample was mounted using glycerol onto the microscope slides. The images were taken at 10x magnification using Olympus CKX53SF inverted microscope (Olympus, Japan). Percentage of alkaline phosphatase positive (ALP+) cells was assessed every 2 days until day 16 of the culture period. images were taken at 10x magnification using Olympus CKX53SF inverted microscope (Olympus, Japan).

Western Blot

Following the viral inoculation, the ALE model was gently washed with ice-cold PBS, cells were lysed in RIPA lysis buffer containing protease inhibitors cocktail (EDTA-free) (Abcam, Cambridge, UK). For western blot, the total protein was quantified by Bradford assay and denatured by boiling at 95°C. An equal amount of protein samples was separated on 15% (w/v) SDS-PAGE gel and then electrically transferred to a 0.45 μm of nitrocellulose membrane (Bio-Rad, US). After blocking with 1×PBS containing 5% (w/v) BSA-Tween (BSA-T), the membrane was then incubated with a specific primary antibody against A29 (Sino Biological, China) and Actin (Merck Millipore, Germany) at 4°C overnight. Then, the membrane was washed three times with 0.1% (w/v) PBS-T and incubated with HRP-conjugated secondary antibody for 1 h at room temperature. The bound antibodies were detected using the Clarity Western ECL Substrate according to the manufacturer's instructions (Bio-Rad, US). Images were obtained using the Gel Doc

XR+ Gel Documentation System (Bio-Rad, US).

Real-time PCR

DNA extraction was conducted using the Genti Advanced Viral DNA/RNA extraction kit and an automatic DNA/RNA extraction machine (GENTi™ 32). Real-time PCR (rt-PCR) reactions were prepared using HotStarTaq DNA polymerase (QIAGEN) and SYBR Green I (Sigma-Aldrich). Quantification of the Mpox gene and the housekeeping gene was performed using primers targeting the TNF receptor gene of Mpox (Mpox TNF) and GAPDH, respectively [6,7]. The primer sequences are provided in Table S3. rt-PCR was carried out using a Rotor-Gene Q (QIAGEN). Results were obtained as cycle threshold (CT) values for each gene.

Assessment of innate immune response and inflammatory mediator using Milliplex and ELISA

To inactivate Mpox-virus within the cell supernatants, the virion isolate was mixed with 10% (v/v) of Triton-X in a 1.5-mL Eppendorf tube to get a final concentration of 0.5% (v/v) before performing the analysis. Levels of IL-1β, IL-6, MIP-1α, and TNF-α in cell supernatants were measured using the customized Milliplex Human Cytokine/Chemokine/Growth Factor Panel for 4 markers (HCYTA-60K-04, Merck Millipore, US). The sample, 100 μL, was used to assay and read using Luminex 200™ prior to analysis with xPONENT version 3.1 software. The diluted standards and quality control were used according to the manufacturer's instructions. On the other hand, the level of galectin-9 in the samples was measured using a human galectin-9 ELISA kit (EH206RB, Invitrogen, US). Galectin-9 was measured by reading the absorbance at a wavelength of 450 nm using a plate reader (BioTek Epoch Plate Reader + Take 3, US). All analytes were determined from six subject samples (n=6) and the data were displayed

in pg/mL.

Assessment of cellular distribution of Mpox using Transmission electron microscopy (TEM) analysis

Transwell™ membrane sample was fixed using a glutaraldehyde fixative solution (2% (v/v) glutaraldehyde and 3.7% (v/v) paraformaldehyde) in 0.2 M PIPES buffer for 3 hours at 4°C. After post-fixation for 1 h in 2% (w/v) osmium tetroxide and dehydration in graded ethanol series (50%, 70%, 80%, 90%, and 100% (v/v)) the samples were embedded in Araldrite resin. Ultrathin sections (50 – 70 nm) were cut using a diamond knife (DiATOME 45° ultra, USA) and stained with 1% (w/v) uranyl acetate and 3% (w/v) lead citrate, sequentially, 10 minutes each. The sample grids were, then, rinsed and dried in a desiccator before they were observed using a JEM-1400 TEM (JEOL, Japan) with an acceleration voltage of 100 kV and magnification at 4,000 – 50,000x.

Transepithelial electrical resistance (TEER)

The epithelial barrier function of epithelial monolayer cultures was determined by measuring the transepithelial electrical resistance (TEER) using the MilliCell-ERS equipped with a pair of chopstick electrodes (Millipore, Bedford, MA, US). TEER was expressed as $\Omega \cdot cm^2$. Briefly, the membrane coated with collagen type-1 of 24-Transwell™ insert (Corning, US) without cells was used as a control while the membrane with cells was used as a test group. To measure TEER at the air-liquid interface, 200 µL of prewarmed fresh serum-free media was added to the apical chamber of the Transwell™ insert and was kept in the cell culture incubator for at least 5 minutes before measuring. TEER value was determined on day 0 as a starting point and was measured again 48 hours post-Mpox virus inoculation. TEER values were averaged from 4-well readout values in order to find the average TEER value per subject sample. Calculations of five independent

experiments (n=5) to obtain values in Ω•cm^2 were made by subtracting the resistance measurement of the blank filter coated with collagen type-1 and multiplying by the area of the monolayer (0.33 cm^2).

References

[1] Chen S. Ultrafast one-pass FASTQ data preprocessing, quality control, and deduplication using fastp. iMeta. 2023;2(2):e107. doi: 10.1002/imt2.107.

[2] Li H, Durbin R. Fast and accurate short read alignment with Burrows–Wheeler transform. Bioinformatics. 2009;25(14):1754–1760. doi: 10.1093/bioinformatics/btp324.

[3] Li H, Handsaker B, Wysoker A, *et al*. The sequence alignment/map format and SAMtools. Bioinformatics. 2009;25(16):2078–2079. doi: 10.1093/bioinformatics/ btp352.

[4] Grubaugh ND, Gangavarapu K, Quick J, *et al*. An amplicon-based sequencing framework for accurately measuring intrahost virus diversity using PrimalSeq and iVar. Genome Biol. 2019;20(1):8. doi: 10.1186/s13059-018-1618-7. doi: 10.1186/s13059-018-1618-7.

[5] Aksamentov I, Roemer C, Hodcroft EB, *et al*. Nextclade: Clade assignment, mutation calling and quality control for viral genomes. J Open Source Softw. 2021;6(67):3773. doi: 10.21105/joss.03773.

[6] Silva SJR da, Kohl A, Pena L, *et al*. Clinical and laboratory diagnosis of monkeypox (mpox): Current status and future directions. iScience. 2023;26(6):106759. doi: 10.1016/j.isci.2023.106759.

[7] Boonhok R, Rachaphaew N, Duangmanee A, *et al*. LAP-like process as an immune mechanism downstream of IFN-γ in control of the human malaria *Plasmodium*

vivax liver stage. Proc Natl Acad Sci USA. 2016;113(25):E3519–E3528. doi: 10.1073/pnas.1525606113.

Tables and figure legends

Table S1. Tissue donor characteristics

**Donor**	**Age**	**Sex**	**Race**
1 (30-08-2023)	71	Female	Asian
2 (06-09-2023)	69	Male	Asian
3 (20-09-2023)	66	Female	Asian
4 (28-09-2023)	55	Male	Asian
5 (04-10-2023)	71	Male	Asian
6 (13-09-2023)	74	Female	Asian
7 (29-11-2023)	71	Female	Asian
8 (22-11-2023)	67	Female	Asian
9 (08-11-2023)	75	Female	Asian
10 (15-11-2023)	51	Female	Asian
11 (20-12-2023)	73	Male	Asian

Table S2. High content analysis detail

**1. Stack processing**	Maximum Projection
**2. Find Nuclei**	Channel: Hoechst 33342<br>ROI: None<br>Method: M<br>- Diameter: 13 µm<br>- Splitting Sensitivity: 0.22

	- Common Threshold: 0.39<br>Output: Nuclei
**3. Find Cytoplasm**	Channel: Alexa 488<br>Nuclei: Nuclei<br>Method: E<br>- Common Threshold: 0.36<br>- Individual Threshold: 0.05<br>Output Population: Cells
**4. Calculate Morphology Properties**	Population: Cells<br>Region: Cell<br>Method: Standard<br>Property Prefix: Cell Morphology
**5. Calculate Intensity Properties**	Channel: Alexa 488<br>Population: Cells<br>Region: Cell<br>Method: Standard<br>Property Prefix: Intensity Cell Alexa 488
**6. Calculate Intensity Properties**	Channel: Alexa 555<br>Population: Cells<br>Region: Cell<br>Method: Standard<br>Property Prefix: Intensity Cell Alexa 555
**7. Select Population**	Population: Cells<br>Method: Filter by Property<br>- Intensity Cell Alexa 555 > 500

	- Cell Morphology > 80<br><br>Output Population: AEC
**8. Select Population**	Population: Cells<br><br>Method: Filter by Property<br><br>- Intensity Cell Alexa 488 > 1500<br><br>- Cell Morphology > 80<br><br>Output Population: Mpox Infected Cell
**9. Calculate Morphology Properties**	Population: Mpox Infected Cell<br><br>Region: Cell<br><br>Method: Standard<br><br>Property Prefix: Mpox Infected Cell Morphology
**10. Select Population**	Population: Mpox Infected Cell<br><br>Method: Filter by Property<br><br>- Number of Nuclei ≥ 2<br><br>Output Population: Multinucleated Cell
**10. Select Population**	Population: AEC<br><br>Method: Filter by Property<br><br>- Intensity Cell Alexa 488 > 1500<br><br>Output Population: A29+ and RAGE+ or SFTPC+
**11. Define Result**	Method: Standard Output<br><br>1. Cell – Number of Objects<br><br>2. AEC – Number of Objects<br><br>3. Cell – Mean Cell Area (μm^2) per Well<br><br>4. Mpox Infected Cell – Number of Objects

	5. Mpox Infected Cell – Mean Cell Area (μm^2) per Well<br>6. Multinucleated Cells – Number of Objects<br>7. A29$^+$ and RAGE$^+$ or SFTPC$^+$ – Number of Objects

Table S3. Primers used in this study

Genes	Forward primer	Reverse primer
Mpox TNF	5'-GGAAAATGTAAAGACAACGAATACAG -3'	5'-GCTATCACATAATCTGGAAGCGTA-3'
GAPDH	5′-ATG GGG AAG GTG AAG GTC G-3′	5′-GGG GTC ATT GAT GGC AAC A-3′

Figure S1. Pro-SFTPC and CAV1 expression kinetics over a 9-day culture period. Immunofluorescent images of cells expressing pro-SFTPC (A - C) and CAV1 (D - F) from Day 3 to Day 9. Confocal images were taken at 10x magnification, scale bar at the bottom. **Green** color represents **Pro-SFTPC**, **orange** color represents **CAV1**, and **blue** color represents **nuclei**.

Figure S2. Genomic analysis of the Mpox isolates from a clinical patient showed the classification of the isolated Mpox within the phylogenetic tree.

Figure S3. Life Cycle of Mpox Clade IIb in the human alveolar epithelium. (1) Binding: Mpox virion penetrates through the lung surfactant layer and binds to unknown receptors on the hAEC. (2) Entry: Mpox can enter the cell through 2 mechanisms either by direct

fusion with the host cell membrane or through micropinocytosis. (3) Escaping from endosomes: Once in the endosome, the virions can release their components into the cell by the fusion between Mpox and endosome membranes. (4) Transportation to the viral factory by microtubules: Viral dsDNA and early viral proteins are transported to the viral factory with the ease of the cellular microtubule network. (5) Viral maturation: Viral DNA begins to replicate as well as Mpox early viral protein synthesis. The formation of Mpox virions starts from the construction of crescent structures that progress into fully mature virions with their first membrane. (6) Transport to trans-Golgi network by microtubule: The newly matured virions are transported to the trans-Golgi network *via* the microtubule for decorating with an additional membrane. (7) Acquisition of double membrane: The transported mature virions will acquire an additional membrane at the Golgi apparatus. (8) Transport to the periphery of the cell by microtubule: The wrapped virions are then transported to the periphery of the cell using the microtubule network. (9) Release and transmission: The matured virions are released from the cell through cell lysis, facilitating host-to-host transmission. Meanwhile, the wrapped virion can be propelled *via* the actin tails from the surface of cell membrane. Viral components and virions can also be transported through TNTs that are generated by microtubules and actin tails.
